# Atrial Fibrillation and Delayed Gastric Emptying

**DOI:** 10.1371/journal.pone.0025499

**Published:** 2011-10-20

**Authors:** Isadora C. Botwinick, R. Joseph Shonkwiler, John Steele, Gary Yu, John A. Chabot

**Affiliations:** Department of Surgery, New York-Presbyterian Hospital, Columbia University Medical Center, Columbia University, New York, New York, United States of America; University of Oxford, United Kingdom

## Abstract

**Background:**

Atrial fibrillation and delayed gastric emptying (DGE) are common after pancreaticoduodenectomy. Our aim was to investigate a potential relationship between atrial fibrillation and DGE, which we defined as failure to tolerate a regular diet by the 7^th^ postoperative day.

**Methods:**

We performed a retrospective chart review of 249 patients who underwent pancreaticoduodenectomy at our institution between 2000 and 2009. Data was analyzed with Fisher exact test for categorical variables and Mann-Whitney U or unpaired T-test for continuous variables.

**Results:**

Approximately 5% of the 249 patients included in the analysis experienced at least one episode of postoperative atrial fibrillation. Median age of patients with atrial fibrillation was 74 years, compared with 66 years in patients without atrial fibrillation (p = 0.0005). Patients with atrial fibrillation were more likely to have a history of atrial fibrillation (p = 0.03). 92% of the patients with atrial fibrillation suffered from DGE, compared to 46% of patients without atrial fibrillation (p = 0.0007). This association held true when controlling for age.

**Conclusion:**

Patients with postoperative atrial fibrillation are more likely to experience delayed gastric emptying. Interventions to manage delayed gastric function might be prudent in patients at high risk for postoperative atrial fibrillation.

## Introduction

Atrial fibrillation and delayed gastric emptying (DGE) are common after pancreaticoduodenectomy (Whipple procedure) [1]. Postoperative atrial fibrillation is associated with increased morbidity and mortality, as well as prolonged hospital stay, which taxes staff and resources [2]. A study of 4181 patients, published in Annals of Internal Medicine, examined the impact of postoperative supraventricular arrhythmia in patients having noncardiac surgery. The most common supraventricular arrhythmia observed was atrial fibrillation; postoperative supraventricular arrhythmia was associated with a 33% increase in length of stay. Patients with postoperative supraventricular arrhythmias were found to be at significantly increased risk of cerebrovascular accident, pulmonary embolism and gastrointestinal bleeding [2]. Its association with gastrointestinal complications, however, has not been extensively studied.

DGE can be a source of distress and discomfort to patients; worse, patients may require total parenteral nutrition (TPN) if their return to normal gastrointestinal function is significantly delayed. Both cardiac rhythm and gastric emptying are mediated by parasympathetic vagal input. Our aim was to investigate a potential relationship between atrial fibrillation and DGE, which, in keeping with ISGPS criteria, we defined as failure to tolerate a regular diet by the 7^th^ postoperative day [3]. To our knowledge, this is the first time such a relationship has been investigated.

## Methods

This study was approved by the Columbia University Institutional Review Board. Requirement of informed consent was waived by the Columbia University Institutional Review Board as this was a retrospective chart review with no direct patient contact. We performed a retrospective chart review of 249 patients who underwent pancreaticoduodenectomy at our institution between 2000 and 2009. We collected: patient demographics, history of cardiac disease, type of resection performed (pylorus-preserving pancreaticoduodenectomy or standard pancreaticoduodenectomy, with or without vascular resection), estimated blood loss, tumor size and histology lymph node status, postoperative complications and postoperative NSAID (ketorolac) use. At our institution, all patients undergoing Whipple procedure also undergo intraoperative placement of a gastrostomy tube (G-tube). This obviates the need for a nasogastric tube, should the patient experience gastroparesis. The institutional protocol for G-tube clamp trials and diet is as described in Table 1.

**Table 1:** G-tube management and diet.

Postoperative day	Plan
0–1	G-tube to gravity and small sips of clears for 24–48 hours
2	6 hour G-tube clamping trials, check for residuals q6 hours
3	If G-tube residuals >250 ml, continue with 6 hour clamping trials. if G-tube residuals <250 ml, then 24 hour clamping trial.
4	IF 24 hour residual < 250 ml, clamp tube and start clear diet with G-tube clamped; open G-tube if patient complains of nausea/vomiting or abdominal pain
5	If tolerating clamping trials, advance patient to regular diet

Data was analyzed with alpha = 0.05 using Fisher exact test for categorical variables and Mann-Whitney U or unpaired T-test for continuous variables. Statistical packages used for the analysis were SAS and GraphPad Prism (v5.0b).

## Results

Approximately 5% of the 249 patients included in the analysis experienced at least one episode of postoperative atrial fibrillation. First line treatment for hemodynamically stable patients in acute need of rate control was generally with beta-blockers or calcium channel blockers. One patient in the atrial fibrillation group sustained a post-operative myocardial infarction.

Patient and tumor characteristics are described in Table 2. Postoperative complications are detailed in Table 3. Median age of patients with atrial fibrillation was 74 years, compared with 66 years in patients without atrial fibrillation (p = 0.0005). Patients with atrial fibrillation were more likely to have a history of atrial fibrillation (p = 0.03). Interestingly, 92% of the patients with atrial fibrillation suffered from delayed gastric emptying (DGE), compared to 46% of patients without atrial fibrillation (p = 0.0007). To determine if age was a confounder of this association, the Breslow-Day test was applied to two 2×2 tables for patient age ≤65 years and age >65 years to test for age as a confounder of this association. This association held true (Breslow-Day test p-value>0.05), suggesting that age does not confound this analysis.

**Table 2:** Factors associated with postoperative atrial fibrillation.

	Atrial fibrillation N = 13	No atrial fibrillation N = 236	P value
Median age	74 (range 63–85) IQR 15.75	66 (range 25–88) IQR 13	0.0005
DGE	12	102	0.0008
Prior atrial fibrillation	3	11	0.03
Male	5	113	ns
Caucasian	10	182	ns
Non-pylorus preserving Whipple	7	150	ns
Estimated blood loss (cc)	1000	1000	ns
Vascular reconstruction	1	43	ns
Median Operative time (minutes)	288.5	352.5	ns
Median positive lymph nodes	0 range (0–10)	0 range (0–16)	ns
Median maximal tumor diameter (cm)	2.9	2.5	ns
Adenocarcinoma	6	146	ns

**Table 3:** Additional postoperative complications.

	Atrial fibrillation13	No atrial fibrillation236	P value
Pancreatic leak	2	12	ns
UTI	1	14	ns
Wound infection	0	17	ns
Death	0	3	ns

Frequency of pancreatic leak, UTI or wound infection was not significantly different between the patients with and without atrial fibrillation. Pancreatic leak was diagnosed clinically as most patients do not have a drain left during surgery and we do not follow drain amylase levels in asymptomatic patients.

## Discussion

### Age and Cardiac History

The incidence of paroxysmal atrial fibrillation in the general population rises sharply with increasing age [4], [5]. Our data are consistent with this finding; older patients in this study had a higher risk of developing postoperative atrial fibrillation. As we expected, prior episodes of atrial fibrillation increased the risk of postoperative atrial fibrillation after Whipple.

### Delayed gastric emptying

We demonstrated a strong correlation between postoperative atrial fibrillation and subsequent delayed gastric emptying. We propose vagal innervation as a common mechanism underlying the relationship between these morbidities. A known subset of atrial fibrillation, so called “vagal paroxysmal atrial fibrillation” is thought to arise from excessive parasympathetic or vagal tone. Patients typically experience episodes of atrial fibrillation at night, or postprandially, when vagal tone is high [6]. Vagal input also affects gastric emptying, as well as visceral pain [7]–[8]. Postoperative patients may therefore have higher levels of vagal tone secondary to postoperative pain and ileus. We hypothesize that, in susceptible patients, this increased level of vagal tone could result in postoperative paroxysmal atrial fibrillation. Postoperative bowel edema and third space sequestration could also cause delayed gastric emptying. When sequestered fluid is mobilized, the rise in intravascular volume may predispose patients to atrial fibrillation [9].

The limitations of this study are the retrospective nature of the data collection. Ideally, the findings we present would be verified by additional prospectively-collected data. Additional exploration of the possible underlying pathomechanism would help further delineate causality. RR interval (respiratory sinus arrhythmia) is the time elapsing between two consecutive R waves in the EKG. RR interval variation is an established, noninvasive measure of baseline parasympathetic tone [10]. One might hypothesize that patients determined to have a higher baseline vagal tone by preoperative EKG, would be more prone to postoperative atrial fibrillation and delayed gastric emptying and thus would derive the most benefit from a therapeutic intervention, such as intraoperative placement of a feeding jejunostomy tube.

There has been extensive research into the etiology of postoperative ileus, with several recent studies implicating an inflammatory field effect. McCarthy [11] found that inflammatory cytokines IL-1, IL-6 and TNF likely mediate gastroparesis in a fasted animal model, while Wehner and colleagues. noted that in a rodent model, mechanical manipulation of the bowel resulted in a similar inflammatory response, which appeared to be macrophage-mediated [12]. Engel et al further refined this concept, demonstrating that the intestinal macrophages are activated by T-helper type 1 cells which can migrate through the bloodstream and induce ileus in areas of the bowel that have not been surgically manipulated [13]; Liebgrets et al noted a similar T-cell effect on ileus in nonsurgical patients with functional dyspepsia [14].

A number of studies have shown that vagal input opposes the inflammatory cascade thought to contribute to postoperative ileus. De Jonge et al demonstrated that vagal nerve stimulation by lipid-rich nutrition or direct stimulation of efferent parasympathetic vagal nerve fibers could prevent postoperative ileus by attenuating this inflammatory response [15]. Pavlov and Tracey further elaborate on the topic with the concept of the “inflammatory reflex”, citing numerous observations in support of an immunomodulatory reflex mechanism in which inflammation triggers a vagally-mediated antiflamatory reaction [16].

Our findings are consistent with prior research. Our patients underwent surgery with a subset experiencing subsequent delayed gastric empyting, likely secondary to an inflammatory response triggered by mechanical manipulation of the bowel. In response to this original inflammatory effect, and likely also to in response to visceral distention from established ileus, an anti-inflammatory vagal response was triggered. This reactive vagal response could have triggered atrial fibrillation in susceptible patients. While large studies are rare, some papers suggest that among patients with paroxysmal atrial fibrillation, between 10–15% [17] have vagal atrial fibrillation. These data suggest that, in the population as a whole, only a small subset of patients are sufficiently susceptible to heightened vagal tone as the precipitating factor in atrial fibrillation; it explains why, of our 114 patients with delayed gastric emptying, only 12 (11%) experienced atrial fibrillation.

As detailed above, we have observed an association between postoperative atrial fibrillation and delayed gastric emptying. Given the retrospective nature of this study, it is difficult to determine causality in the relationship between atrial fibrillation and delayed gastric emptying. However, amongst our patients with atrial fibrillation, most developed the arrhythmia in the first five days after surgery (median day three). Delayed gastric emptying, by definition, is diagnosed after the seventh day of diet intolerance. From a purely chronological perspective, we can thus state that patients with postoperative atrial fibrillation are more likely to experience delayed gastric emptying, although as discussed earlier in the paper, from etiological perspective, the physiological changes that result in delayed gastric emptying may be the cause of atrial fibrillation, even before delayed gastric emptying is diagnosed. Interventions to manage delayed gastric function, such as the intraoperative insertion of a gastrostomy or feeding jejunostomy tube might be prudent in patients at high risk for postoperative atrial fibrillation.
